# Antimicrobial Effects of Dental Luting Glass Ionomer Cements on *Streptococcus mutans*

**DOI:** 10.1155/2014/807086

**Published:** 2014-03-23

**Authors:** Sina Klai, Markus Altenburger, Bettina Spitzmüller, Annette Anderson, Elmar Hellwig, Ali Al-Ahmad

**Affiliations:** Department of Operative Dentistry and Periodontology, University Hospital and Dental School, Albert-Ludwigs-University, Hugstetter Straße 55, 79106 Freiburg, Germany

## Abstract

*Objective*. To reduce secondary caries, glass ionomer luting cements are often used for cementing of indirect restorations. This is because of their well-known antimicrobial potential through the release of fluoride ions. The aim of this *in vitro* study was to investigate the antimicrobial effect of five dental luting cements which were based on glass ionomer cement technology. *Methods*. Five different glass ionomer based luting cements were tested for their antimicrobial effects on *Streptococcus mutans* in two different experimental setups: (i) determination of colony-forming units (CFUs) in a plate-counting assay; (ii) live/dead staining (LDS) and fluorescence microscopy. All experiments were conducted with or without prior treatment of the materials using sterilized human saliva. Antimicrobial effects were evaluated for adherent and planktonic bacteria. Bovine enamel slabs (BES) were used as negative control. BES covered with 0.2% chlorhexidine (CHX) served as positive control. *Results*. Each of the tested materials significantly reduced the number of initially adhered CFUs; this reduction was even more pronounced after prior incubation in saliva. Antimicrobial effects on adherent bacteria were confirmed by live-dead staining. *Conclusion*. All five luting cements showed an antimicrobial potential which was increased by prior incubation with human saliva, suggesting an enhanced effect *in vivo*.

## 1. Introduction

Under physiological conditions the oral cavity accommodates a variety of different bacteria living in an ecological system [1–4]. However, disturbances or changes in conditions can induce shifts of benign microflora towards pathologies such as caries.

Microorganisms in the oral cavity are organized as a biofilm [5]. Dental biofilm, called plaque, is a histologically structured, dense, felted mass of bacteria in their self-produced extracellular polymeric matrix [6, 7].

The ability to form this matrix modifies the local environment, which in turn enables life even for microorganisms which are not usually viable within the oral cavity. Such modifications involve pH-value, redox potential, and possible gene transfer [8, 9]. A gradient of decreasing bacterial metabolism and increasing doubling rate is created from the top of the biofilm to the bottom. This gradient plays a key role in antibiotic resistance and presents a selective advantage for biofilm microorganisms [6, 7, 10].

Undisturbed bacterial colonization and growth within the oral cavity lead to biofilm induced caries, gingivitis, and periodontitis [11].* Streptococcus mutans* is the main etiological agent of dental caries caused by dental biofilm. It adheres to a proteinous layer, the acquired pellicle, already present on the enamel. The pellicle consists of preadsorbed salivary proteins, lipids, and glycolipids [12, 13].

Since initial adhesion is the first step in biofilm development, research has focused on strategies to prevent initial microbial colonization and subsequently reduce or inhibit biofilm formation. Avoiding initial colonization is merely impossible, therefore it is necessary to develop other methods to prevent caries. Secondary caries is a major problem in restoration dentistry which develops at leaky crown margins or insufficient other restorations. Cements with antimicrobial potential may be worthwhile in preventing secondary caries. Teeth prepared for indirect restoration expose much more dentine than healthy teeth, a fact that also must be taken into account when considering caries progress. Dentine has a different histological and micromorphological structure compared to enamel. It is perforated with dentine tubes which allow faster progression of the caries into the pulp.

It has been proposed that glass ionomer cements may decrease caries demineralization at margins of restorations. Despite their poor characteristics with regard to bending strength, esthetic, and surface polishability [14, 15], they have one prominent advantage: fluoride is released over a long period of time [16]. Furthermore, glass ionomer cements were shown to be rechargeable with fluoride ions* in vitro* [17–19]. This characteristic makes these materials popular as luting agents, because fluoride can hamper demineralization and promote remineralization of dental hard tissue.

The aim of this study was to investigate whether there are any effects from five different glass ionomer luting materials on the initial adhesion of* S. mutans*. Additionally, the effect of these materials on planktonic bacteria was studied in parallel. Three different approaches and methods were used to determine the effect of hardened cements on* S. mutans* with and without prior storage in sterile human saliva for one week. The hypotheses we tested were that glass ionomer cements which exhibit an antibacterial effect.

## 2. Materials and Methods

### 2.1. Bacterial Strain and Bovine Enamel Specimens

*Streptococcus mutans* DSM 20523 was maintained routinely with weekly subculturing on Columbia Blood Agar (CBA) plates. Long-term storage was conducted at −80°C in basic growth medium containing 15% (v/v) glycerol as described in detail elsewhere [20].

Bovine enamel slabs (BES) used as control were prepared as described in several previous* in situ* studies [20, 21]. Standardized BES of homogenous quality, large surface area, and chemical properties similar to human enamel were obtained for the negative and positive control [22].

Bovine incisors from BSE-free and freshly slaughtered cattle were used to prepare BES with a diameter of 5 mm and a height of 0.35 mm as described by Al-Ahmad et al. [20, 21]. The final grinding of the bovine enamel was conducted on a grinding machine (Knuth-Rotor-3, Streuers, Willich, Germany) using wet sand paper of 1200, 2400, and 4000 grids in decreasing order of grain sizes. The surface of the enamel specimens was then controlled by impinging light microscopy (Leica Wild M3Z, Germany). Prior to usage, the bovine enamel slabs were sterilized by ultrasonication for 3 min in 2% sodium hypochlorite (NaOCl) and 3 min in 70% ethanol. Subsequently, the samples were washed twice and stored for 48 hours in sterile distilled water. Sterility was tested by aerobic and anaerobic cultivation on CBA. Bovine tooth samples covered by 0.2% chlorhexidine (CHX) were used as a positive control for antimicrobial effects.

### 2.2. Tested Materials

Two commercially available glass ionomer luting cements GC Fuji I (GC) and 3M ESPE Ketac Cem Easymix (3M) (DENTSPLY DeTrey GmbH, Konstanz, Germany) were used. Additionally, three experimental glass ionomer cements called A, B, and C were also investigated. The main components of all three materials were zinc-glass and poly(acrylic acid) (PAA). Material A contained zinc tartrate and material C contained silver-containing glass as additives.

Samples in the shape of frustums were prepared according to the manufacturer's procedures (DENTSPLY DeTrey GmbH, Konstanz, Germany) and tested in triplicates. Each test was conducted two times. Samples were homogenously sized to standardize the surface and volume of the specimens. The hardened samples were ground plane parallel on sand paper with a grain size of 1200, 2400, and 4000 grids in decreasing order of grain size to obtain a homogeneous surface. Afterwards, they were disinfected in 70% ethanol and immediately washed twice in distilled water prior to usage.

### 2.3. Determination of the Log Growth Phase of* Streptococcus mutans*

To ensure that log phase bacteria were used, a growth curve was determined for the strain used in the study.* Streptococcus mutans* (DSM 20523) was grown overnight at 37°C under aerobic conditions with 5% CO_2_ in tryptic soy broth (TSB). The overnight culture had an OD_595_ of 1.2. Then, 100 *μ*L of the overnight culture were inoculated in different culture tubes with 10 mL TSB. The growth of the strain was measured hourly over a total time period of nine hours. OD_595_ nm and CFUs were determined after conducting an appropriate dilution series in 0.9% sodium chloride solution (NaCl). Based on the resulting growth curve, only log phase bacteria were used to study possible inhibition effects of the luting cements on initially adherent bacteria as described below.

The lag growth phase was observed during the first 4 hours after inoculation. The log growth phase started after 4 hours, and after 7 hours* S. mutans* it reached the stationary phase.

For each experiment bacteria which were grown for 5 hours after inoculation with overnight culture were used. This ensured that only viable bacteria from the log growth phase were influenced by the tested materials.

### 2.4. Determination of Colony Forming Units (CFUs)

To determine the number of colony forming units, 200 *μ*L of the overnight culture was inoculated in 10 mL TSB. After 4 hours of incubation at 37°C with shaking, the resulting logarithmic phase cells were used at a concentration of 10^6^ CFU/mL to investigate any effect the luting cements might have on initial adhesion and on the planktonic bacteria. For this purpose, bacteria were centrifuged at 2000 g for 10 min and washed in 0.9% NaCl. The bacterial pellet was resuspended in the same volume of 0.9% NaCl, and the CFUs were determined by plate count method before testing the materials.

Three samples prepared as described above from each material were placed in sterile 1 mL tubes (Eppendorf, Germany). Subsequently, 300 *μ*L of the bacterial suspension was added to each tube and the samples were incubated at 37°C for two hours with constant swirling. After the incubation period 1 mL of the bacterial suspension was removed from the tube and serially diluted up to 10^−3^ to measure the count of planktonic bacteria. One hundred *μ*L of each dilution was then streaked on CBA plates. The CBA plates were incubated at 37°C for 3 days under aerobic conditions and 5% CO_2_ (capnophilic conditions). The CFUs were counted using the Gel Doc EQ Universal Hood (Bio-Rad Life Science group, Hercules, USA).

The frustums remaining in the sample tubes were rinsed twice with 0.9% NaCl to remove nonadherent cells and were subsequently transferred to sterile tubes with 1 mL 0.9% NaCl, vortexed, and then treated for 30 s in an ultrasonic bath on ice to desorb the microorganisms from the surface of the material. This solution was serially diluted up to 10^−3^ in physiological saline and subsequently the plate count was determined as described above for the planktonic bacteria.

For each of the materials and the controls studied, the experiment was conducted twice without prior incubation in sterile human saliva and twice with the materials having been previously incubated for one week in centrifuged, stimulated, and sterile filtered human saliva from a healthy volunteer (nonsmoker, no serious illnesses, no mouth rinses for two weeks, and no antibiotics in the last 3 months).

### 2.5. Live/Dead Staining (LDS)

After incubation of the materials with* Streptococcus mutans* for two hours, the cement frustums were washed with 0.9% NaCl and placed in multiwell plates with 1 mL 0.9% NaCl. The samples were treated with BacLight stain L7007 (Molecular Probes L7007, Invitrogen Ltd., Paisley, UK) which contains two fluorescent dyes, SYTO 9 and propidium iodide. Intact cell membranes are selectively permeable for SYTO 9 (green stain) which can also enter disrupted cells, but not for propidium iodide (red stain). Thus, intact (viable) cells are stained green, while cells with disrupted membranes are selectively stained red. Afterwards the cement samples were placed on object slides and counted using epifluorescence microscopy (Axioskop 2 plus, Zeiss, Oberkochen, Germany). A total of ten different cement locations per specimens was analyzed for every experimental period. This procedure was repeated twice, so that an average vitality of* S. mutans* could be determined. Images were taken with an AxioCam HRC (Zeiss, Oberkochen, Germany), using the KS 300 3.0 software.

### 2.6. Statistical Analysis

Statistical analysis was performed by ANOVA followed by the Tukey's test (IBM SPSS statistics 19.0). The level of significance was *P* < 0.05. *P* values of less than 0.001 were considered highly significant.

## 3. Results

### 3.1. Antimicrobial Effects of the Materials without Prior Storage in Sterile Human Saliva

**Figure 1:**
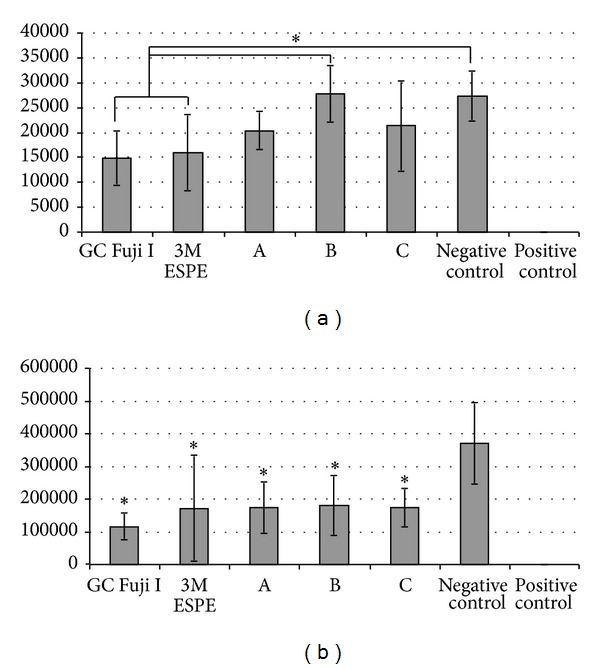
Bacterial count [CFU/mL] of adherent (a) and planktonic (b) bacteria after 4 h incubation of the different ionomer glass cement materials with log phase* Streptococcus mutans*. The *y*-axis shows the CFU/mL.

Figure 1(a) shows the adherent CFUs after incubation of the materials without pretreatment with human saliva. After 2 hours of incubation with log phase* Streptococcus mutans* cells, the materials GC Fuji I and 3M ESPE Ketac Cem Easymix showed a significantly reduced number of CFUs (*P* < 0.05) when compared pairwise to the negative control. This effect was not shown for all experimental glass ionomer materials (A, B, and C) tested. Moreover GC and 3M showed a significant reduction in the number of CFUs when compared to the experimental material B (*P* < 0.05).

Figure 1(b) shows the planktonic CFUs after incubation of the materials without pretreatment with human saliva. All materials showed significantly reduced planktonic CFUs (*P* < 0.05) compared to the negative control. No significant differences among the materials were detected. The bovine enamel slabs covered with chlorhexidine (CHX) and used as positive control revealed a 100% reduction of CFUs.

### 3.2. Antimicrobial Effects of the Materials after Storage in Sterile Human Saliva

**Figure 2:**
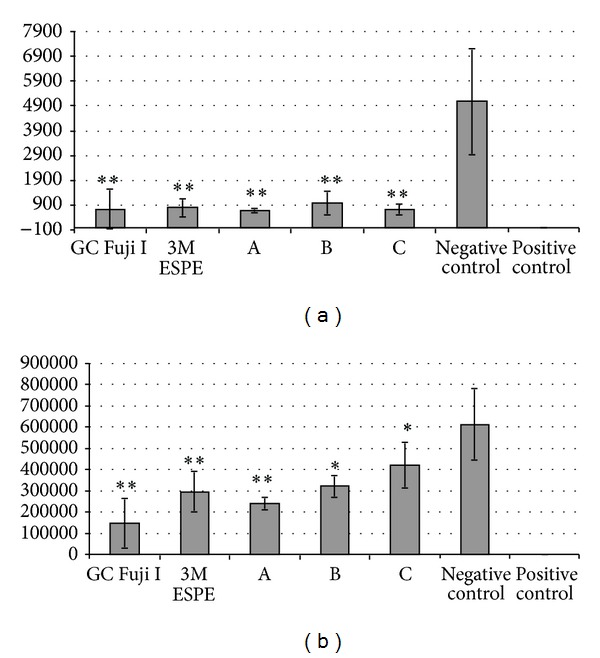
Bacterial count [CFU/mL] of adherent (a) and planktonic (b) bacteria after 4 h incubation of the different ionomer glass cement materials with log phase* S. mutans*. The materials were deposited for one week in sterile human saliva before testing. The *y*-axis shows the CFU/mL.

After 2 hours incubation with log phase cells of* Streptococcus mutans* all materials showed a highly significant reduction of CFUs of adhered bacteria compared to the negative control (*P* < 0.001). No differences among the materials were detected (Figure 2(a)).

Figure 2(b) shows the planktonic CFUs after incubation of the materials with previous storage in human saliva. The materials GC, 3M, and A showed a highly significant reduction in the number of CFUs compared to the negative control (*P* < 0.001). Materials B and C showed a significant reduction in the number of CFUs compared to the negative control (*P* < 0.05).

The positive control (bovine tooth samples with CHX) produced valid results and no living cells were detected.

### 3.3. Live Dead Staining

**Figure 3:**
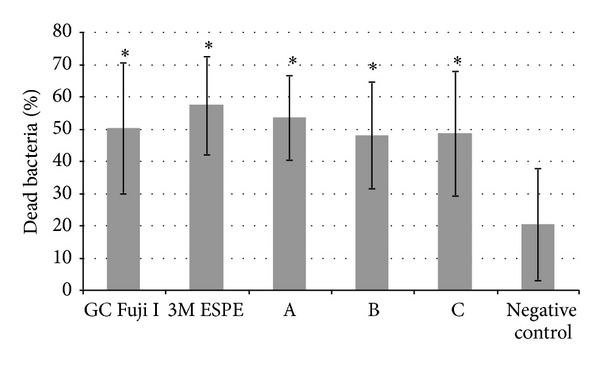
Dead adherent bacteria on the different ionomer glass cement materials in percent after live/dead staining. The materials were incubated with* Streptococcus mutans* for 2 h.

**Figure 4:**
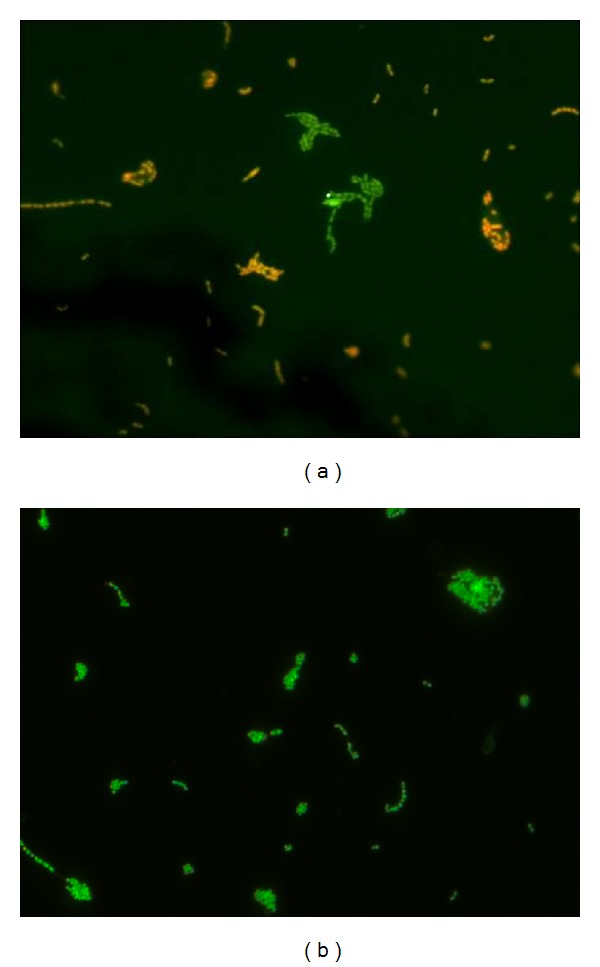
Visualization of adherent bacteria (*Streptococcus mutans*) using epifluorescence microscopy and live/dead staining (1000-fold magnification). Green fluorescent cells depict active bacteria, whereas red fluorescent cells show dead ones. (a) Glass ionomer material; (b) negative control (bovine enamel).

Each material showed a significant antimicrobial effect compared to the negative control (*P* < 0.05). However, pairwise comparisons yielded no statistically significant differences among the materials (Figure 3). In Figure 4, exemplary epifluorescence microscopy images of adherent bacteria on a glass ionomer material (A) and on the negative control (B) are shown. On the glass ionomer material, more dead cells (red) could be observed, whereas on bovine enamel, more viable bacteria could be counted (green cells).

## 4. Discussion

Today secondary caries remains one of the principal reasons for the limited longevity of indirect restoration [23], a serious problem which causes additional costs and is unpleasant for the patient.

Luting cements with antimicrobial potential may protect teeth from secondary caries. Modern glass ionomer cements are suggested to obtain antimicrobial efficacy. Additionally, they release fluoride ions, which help to remineralize initial carious lesions [15–17] and hamper the progression of dental caries [24].

The results of this study demonstrate that each of the tested glass ionomer materials tended to have an antimicrobial effect against* S. mutans*. This is in accordance with previous studies which documented that glass ionomer cements can reduce the number of* S. mutans in vitro* and* in vivo* [18, 25, 26]. The two materials GC Fuji I and 3M ESPE Ketac Cem Easymix seem to have the highest antimicrobial effect. Nevertheless, the three prototypes also seem to be effective against* S. mutans*, with comparable potential. The following tendency was noticed: material A seems to be slightly more effective in its reduction of the CFUs of adhered and planktonic bacteria than material C and material B (Figures 1(a), 2(a), and 2(b)). Material B seems to be the least effective. This tendency could be due to the different additives of material A (Zn-tartrate) and C (silver-containing glass) compared to glass ionomers without additives (material B). Shashibhushan et al. [27] reported that zinc ions released by glass ionomer cements can interfere with substrate transport into the cell and so block important enzyme functions. Yet the most obvious explanation for the observed antimicrobial effects of the cement materials could be the release of fluoride as previous authors have suggested [17–19]. Koo et al. [28] indicate that fluoride ions are mostly bacteriostatic. However, under certain circumstances such as a high concentration, fluoride ions can also generate a bactericidal effect. Furthermore, other ingredients should also be taken into consideration as pointed out by Geurtsen [29].

The determination of CFUs also revealed that storage in sterile human saliva significantly increased the antimicrobial effect of each of the materials. This is in accordance with a previous study of Saku et al. [26] who attributed this effect to the inherent antimicrobial substances found in human saliva. These substances could enhance the antimicrobial potential of the cements so that this effect is reinforced after incubation in saliva. Otherwise, it could be assumed that the enhancement of the antimicrobial effects after storage in saliva might be caused by diffusion changes of antimicrobial substances which are released by the materials themselves. This could lead to an enrichment of antimicrobial substances on the material surface.

The observed enhancement of the antimicrobial effect after incubation of the materials in human saliva leads to the expectation that an* in vivo* influence on oral bacteria could be possible. Such an antimicrobial effect could become manifest in two different ways. On the one hand, substances released by the cements could influence bacterial metabolism, which in turn would induce a decrease in the growth rate. This effect is called bacteriostatic. On the other hand, cells could be killed by these substances, reducing the number of initially viable cells. This effect is called bactericidal.

The storage in saliva leads to the development of the acquired pellicle which forms a proteinous coat around the cements within a short time [12]. Previous authors showed that bacteria can adhere to the lipids and glycolipids of the acquired pellicle and induce biofilm formation in order to protect themselves from harmful substances in their surroundings by modification of their properties [8]. It might be possible that this bacterial resistance is formed immediately after initial adhesion, so that the adherent bacteria are protected from the antimicrobial substances which the materials release. On the other hand, according to our observations, we can assume that the acquired pellicle could also enrich antimicrobial substances released by the materials, since human saliva enhanced the effects of the tested materials.

A critical factor in the CFU experiment is that sonication during sample treatment could produce flocs. Each floc originally contains a number of single bacteria, which introduces a bias toward CFU counts which are too high [13]. However, Al-Ahmad et al. [21] showed that pure sonication generally has no influence on bacterial vitality.

The results of the live/dead staining confirmed the above mentioned antimicrobial effect. Each of the materials showed a significant antimicrobial effect compared to the negative control when using this technique. In contrast, the antimicrobial effect between the particular cements was not significantly different. Overall, each of the tested materials had a similar and comparable antimicrobial potential.

Upadhyay and Rao [30] indicated that it is impossible to avoid microleakages when using these materials in the oral cavity. The results of this study suggest that such microleakages enhance the protecting effect of glass ionomer cements, since the materials are permanently exposed to human saliva.

There are different factors which emphasize the need for further investigation to confirm the results presented in this study. Chemical tests to measure fluoride concentration and material composition in general are needed to detect any further potentially antimicrobial ingredients. Although the tested samples were manufactured in the same way, differences regarding surface structure, charge, fluoride ion release, and composition can lead to differences in their effects on* S. mutans in vitro* [31–34]. Factors such as the continuous flow of saliva, fluctuation in pH or ion concentration, and alteration of the surface could interfere with the effect of the materials* in vivo* [35, 36]. Furthermore, the development of the acquired pellicle follows different patterns within the oral cavity [7]. Therefore,* in situ* studies using splint models as described by Al-Ahmad et al. [37] could be conducted to gain more information.

## 5. Conclusions

Each of the cement materials tested showed antimicrobial activity against* Streptococcus mutans in vitro*. This study shows for the first time that human saliva enhances the antimicrobial potential of glass ionomer cements* in vitro*.
